# Association between urbanicity and physical activity in Mexican adolescents: The use of a composite urbanicity measure

**DOI:** 10.1371/journal.pone.0204739

**Published:** 2018-09-27

**Authors:** Maria E. Hermosillo-Gallardo, Russell Jago, Simon J. Sebire

**Affiliations:** Centre for Exercise, Nutrition & Health Sciences, School for Policy Studies, University of Bristol, Bristol, United Kingdom; TNO, NETHERLANDS

## Abstract

**Purpose:**

In Mexico, 39.5% of adolescents do not meet the World Health Organisation’s physical activity guidelines. Urbanicity is a potential correlate of physical activity. The aim of this study was to examine the associations between different aspects of urbanicity and adolescents’ physical activity.

**Methods:**

Participants were 4,079 Mexican adolescents aged 15–18 from Mexico City and Oaxaca, Mexico. Data was collected between February and June 2016. Multiple imputation of missing data was implemented after confirming values were missing at random. Multivariable regression models examined associations between five domains of self-reported physical activity: 1) moderate-to-vigorous physical activity, 2) sports activities, 3) leisure time activities, 4) Physical Education class at school, 5) active commuting to school; and a composite measure of urbanicity and its seven sub-scores: 1) demographic, 2) economic activity, 3) built environment, 4) communication, 5) education, 6) diversity and 7) health services. Multivariable regression models were adjusted for parents’ education and participants’ age.

**Results:**

Urbanicity was positively associated with activity spent in Physical Education class. The association between urbanicity and sport activities depended on state context. Communication-based urbanicity was negatively associated with leisure physical activity and active commuting. Population density was positively associated with active commuting.

**Conclusion:**

Urbanicity is associated with adolescents’ physical activity in Mexico. Findings were largely consistent between Mexico City and Oaxaca and highlight the value of examining urbanicity as a multidimensional construct.

## Introduction

Physical activity is associated with reduced risk of developing non-communicable diseases [1]. The latest National Health and Nutrition Survey 2016 in Mexico reported that 39.5% (30.1% males & 48.8% females) of adolescents between 15 and 19 years old do not meet the World Health Organisation’s guidelines for physical activity of at least 60 minutes of moderate-to-vigorous physical activity (MVPA) per day [1].

Overweight and obesity are a global health issue [2]. Mexico has one of the highest prevalence of obesity and overweight (combined prevalence 72.5%, 95% CI = 70.8 to 74.3) in the world [3]. The prevalence of overweight and obesity amongst adolescents is 26.4% and 12.8% respectively for girls, and 18.5% and 15% respectively for boys [4]. Previous research has found that physical activity is a viable strategy to treat and prevent overweight and obesity in adolescents [5]. Authors from a systematic review concluded that 155–180 minutes per week of MVPA is effective for reducing body fat in overweight children and adolescents [6], while in another systematic review in Latin America, combined interventions targeting nutrition and physical activity were most successful for the prevention of overweight and obesity in young people aged five to 17 years old [7].

Urbanicity and environmental features (e.g., speed of traffic, number of pedestrian crossings and sidewalks) have been identified as potential correlates of physical activity [8]. Urbanicity is defined as “the impact of living in urban areas at a given point in time … the presence of conditions that are particular to urban areas or are present to a much greater extent than in non-urban areas” [9]. The study of urbanicity is particularly interesting in low and middle income countries such as Mexico due to the rapid rate of urbanisation and migration of people from rural to urban areas [10]. There is strong evidence that urbanicity is associated with increased odds of adults in India being classed as having low physical activity (men: OR: 3.26; 95% CI = 2.5 to 4.3 and women: OR: 4.13; 95% CI = 3.0 to 5.7) [11]. Similarly, the physical activity level (PAL = total energy expenditure/basal metabolic rate) of adults dwelling in urban areas of Papua New Guinea was lower (PAL = 1.63±0.19, sedentary or light activity lifestyle) than those in rural areas (PAL = 1.88±0.26, active or moderately active lifestyle) (p<0.01) [12]. Further, overall urbanicity in Mexico accounted for a mean decrease of moderate physical activity between 10 and 17.6 minutes per week, and education and diversity urbanicity sub-scores were negatively associated with vigorous physical activity amongst adults [8]. In a study with Kenyan adolescents, urban habitants reported lower MVPA (18 minutes per day in men & 25 minutes per day in women (p<0.05)), than those living in a rural environment [13]. Urbanicity involves the study of many components (i.e., built environment, presence of communication media, availability of health services and population density). Some of these elements have been found in the literature to have a positive effect in adolescents’ physical activity. A meta-analysis found that features from the built environment (paved roads, walking paths, public lighting, traffic lights, street connectivity) had a positive effect on adolescents’ MVPA [14]. Moreover, the presence of streets and pavements has been associated with greater odds of being active among adolescents [15] and the perception of walkable destinations and the presence of open space had been associated with meeting recommendations for walking [16, 17]. Even though these elements of urban environment have been positively associated with physical activity in adolescents, there are other elements of urbanicity that have been found detrimental for health. For example, the presence of television in the bedroom has been associated with an increase of odds of having a large waist circumference and high levels of fat mass [18]; and residential density has been positively associated with adolescents’ obesity [19]. The complexity of the association between urbanicity and physical activity might be a reason why people living in highly urbanised areas, who possibly have better access to pavements, cycling paths and recreation areas still have los physical activity. This evidence suggests that the urban environment has an important role in physical activity levels in developing countries such as Mexico. However, currently, there is a lack of evidence on whether urbanicity is associated with adolescents’ physical activity in Mexico.

Urbanisation, as an environmental factor, is one of the fundamental parts of Socio-ecological models. Socio-ecological models, introduced in the 1970’s, address the understanding of the relations between various personal and environmental factors; focusing in social, institutional and cultural contexts of people-environment relations [20]. The most recognized socio-ecological model is the Ecological Model for Human Development from Urie Bronfenbrenner, in which, in order to understand human development, the ecological system (microsystem, mesosystem, exosystem, macrosystem, chronosystem) needs to be taken into account. In 2006, Sallis, J.F. adapted Bronfenbrenner’s levels of behaviour change to active living [21]. The study or urbanisation overlaps with the exosystem ring posed by Bronfenbrenner and its equivalent “Behaviour Setting” from Sallis’ Ecological Model.

A number of different approaches have been used to measure urbanisation. At the simplest level, these approaches have dichotomised locations as urban or rural based on population size/density, economic activity or size of the city [13]. These measures are limited as they only consider basic aspects of urbanisation and fail to quantify other contributory factors, such as the number of paved roads, presence of communication media or proximity to markets which may be associated with physical activity. More recently, several composite measures have been developed which assess broader urbanisation [22–24]. These measures are tailored to the political and socioeconomic situation of a country, allowing the assessment of urbanicity over time and across different environments through multi-item scales (e.g., population size, population density, communication, transportation, educational facilities, health services, markets, housing, diversity, sanitation, & built environment). Recent research in Mexico examined the association between physical activity and urbanicity using the scale by Novak et al., in this study, physical activity was negatively associated with population size/density, economic activity, communication and diversity sub-scales of urbanicity [8]. However, this study used state-level measures of urbanicity that may fail to quantify individuals’ immediate environment, which is likely to have a stronger influence on their physical activity.

The aim of this study was to examine the association between multidimensional local-level urbanisation and the MVPA, sport activities, leisure physical activity, time spent in Physical Education (PE) class and active commuting to school of adolescents in two states of Mexico.

## Methods

### Design

The study used a cross-sectional design. Objectively-assessed urbanicity scores were derived from national statistics. Adolescent physical activity was self-reported. Data were collected in Mexico City and Oaxaca between February and June 2016. Ethical approval was obtained by the Research Ethics Committee of University of Bristol and the relevant permits and approvals for foreign researchers were obtained from the Ministry of Education in Mexico. Also, written informed consent was obtained from all participants. No approval was needed from parents or guardians of the adolescents as the questionnaire did not cover sensitive topics and adolescents between 15 and 18 years old were considered, by the two previously mentioned organisations, old enough to decide themselves whether or not to participate.

### Recruitment of schools & participants

**Fig 1:**
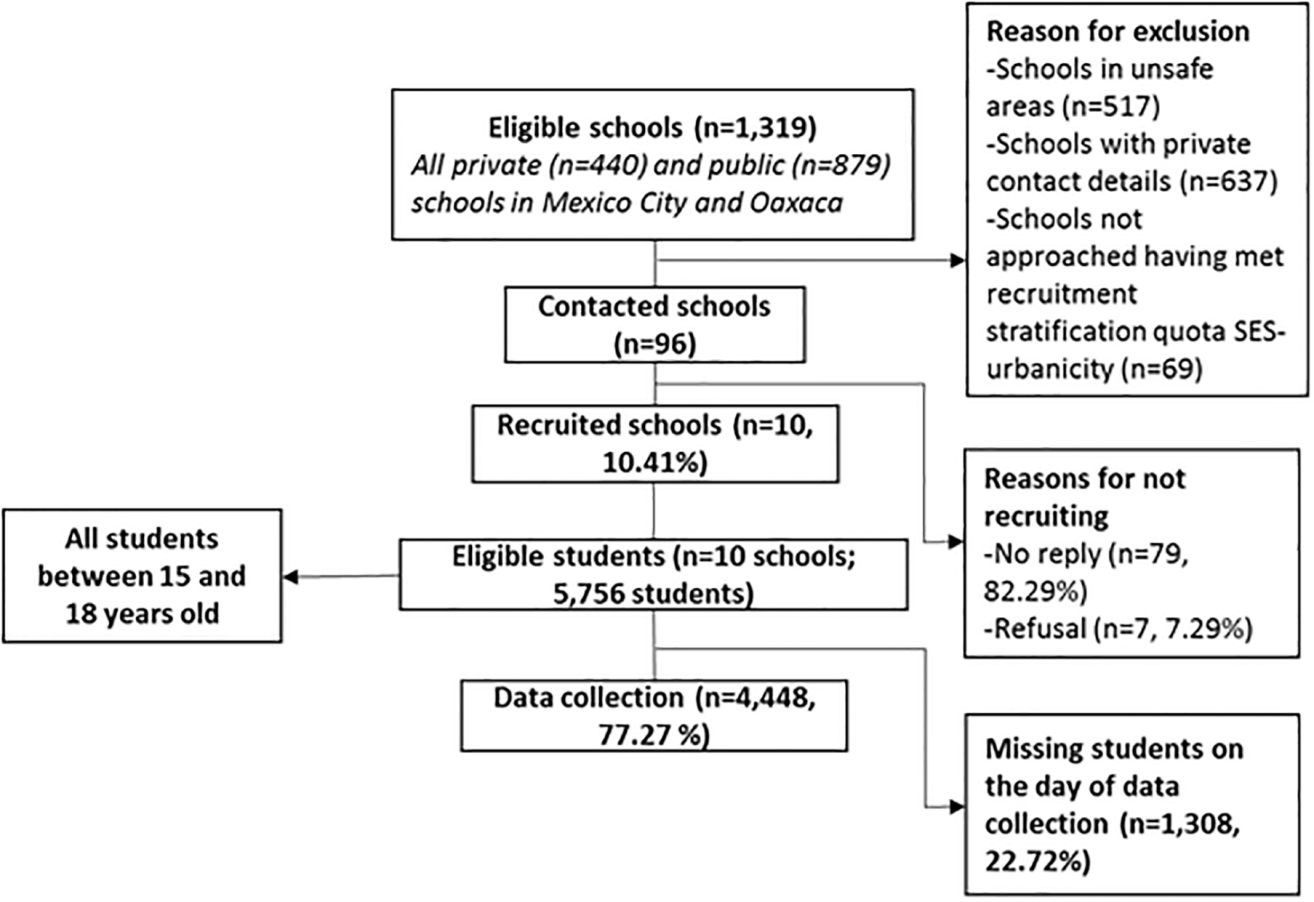
Eligibility criteria.

Based on previous research, Mexico City and the state of Oaxaca were chosen due to their varied urbanicity level (Mexico City: 58.16 vs. Oaxaca: 39.70, on a zero to 70 scale) in which population size/density, economic activity, built environment, communication, education, diversity and health services were the components used to determine urbanicity [8]. Mexico City is the capital of Mexico and has a population size of 8,918,653 in an area of 1,495 km2, with an annual population growth rate of 0.3%. The state of Oaxaca is one with the highest amount of indigenous people in Mexico (65.7% vs. 8.8% in Mexico City) and has a population size of 3,967,889 in an area of 93,757 km^2^, with an annual population growth rate of 0.9 [25]. A list of public schools in both states was provided by the Ministry of Education in Mexico and school sampling was stratified by socio-economic status (SES) and urbanicity level. Each municipality from both states was allocated to a SES tertile (low, medium or high) according to the National Institute of Statistics and Geography from Mexico and then each municipality in the SES categories was given a urbanicity level (low, medium, high) based on previous research [8]. Schools in municipalities were identified and fifteen schools from each SES-urbanicity combination were sampled giving 1,440 potential schools for each state. From that list, 1,319 schools were eligible (with students between 15 and 18 years), 517 were excluded for being in areas deemed unsafe for data collection, 637 for having private contact details and 69 were not approached having met the recruitment stratification quota (Fig 1). Ninety-six schools were contacted; 79 did not reply, seven refused and 10 schools agreed to participate. Data were collected from six schools from Mexico City (n = 1,918) and four schools from Oaxaca (n = 2,530).

Pupils were recruited within schools where the researcher gave all eligible and present pupils an information sheet and a consent form. No consent from parents or guardians was needed as the questionnaire did not cover sensitive topics and previous approval from the Ministry of Education was granted. Consenting participants were asked to compete a questionnaire to report demographic factors, location, environmental factors, physical activity, and sedentary activity during free time as described below. The researcher was available to address questions.

### Assessment of physical activity

Physical activity was measured using the Youth Physical Activity Questionnaire (Y-PAQ), a 7-day recall tool which assesses the duration and frequency of a range of activities performed during weekdays and weekends, school time and leisure time, plus time spent commuting and being sedentary [26]. Participants reported the duration (minutes) and frequency (times per week) of a list of activities which were grouped in five domains of physical activity: 1) Moderate-to-vigorous physical activity (any activity with a metabolic equivalent ≥ 4 kcal·kg−^1^·h−^1^) [27], 2) sports activities (e.g., baseball, football, gymnastics, swimming), 3) leisure time activities (e.g., bowling, roller-skating, playing with pets), 4) Physical Education (PE) class at school, and 5) active commuting to school (walking or cycling). Detailed methods of the physical activity classification are found on S1 Table. The validity of the scores derived from the Y-PAQ have been demonstrated through correlations with objective measures of energy expenditure [28] and accelerometery [29] among adolescents. The Y-PAQ scores have also demonstrated acceptable internal reliability for MVPA in adolescents^22^.

### Urbanicity assessment

**Fig 2:**
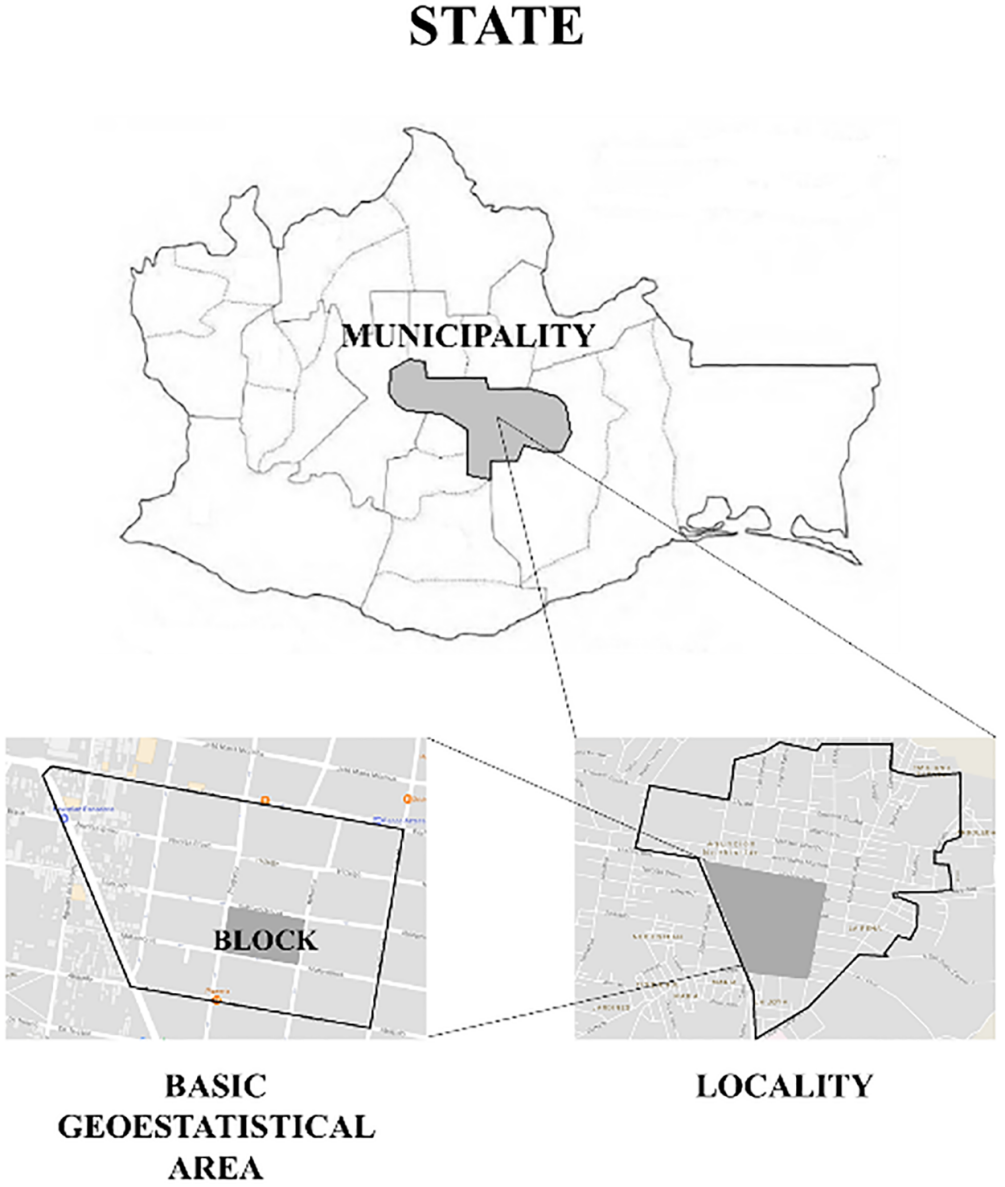
Geographical division of a state.

Location information (home postcode, street name, delegation/municipality and state) was used to allocate each participant within a Basic Geostatistical Area (BAGA) from the CENSUS 2010. A BAGA is a geographical unit defined by the Mexican Government as “a group of blocks (generally between one and 50) delimited by streets and avenues. In rural areas, a BAGA is a territorial extension delimited by paths, rivers/streams, railroad tracks and canyons” [30]. A group of BAGAs form a locality and a group of localities form a municipality within a state (Fig 2).

Urbanicity scores for each BAGA were estimated using data from the National Institute of Statistics and Geography (INEGI), the Public Education Department (SEP), the National Council of Politics and Social Development in Mexico (CONEVAL), and the urbanicity scale developed by Novak et al [24]. The urbanicity scale comprises seven sub-scores: 1) Demographic (people per unit area living in this locality), 2) Economic Activity (proportion of people involved in agriculture as a primary occupation), 3) Built Environment (amount of paved or unpaved roads, sewage services, electricity service), 4) Communication (proportion of houses with television, mobile phone, public internet, public phone), 5) Education (educational facilities per locality, primary school, university, average education of mothers in community), 6) Diversity (variance in housing quality index, variance in mother’s education) and 7) Health Services (health facilities available, health centres, dispensaries/pharmacies, health workers available, village health worker). Each of the seven domains is scored from zero to 10. Due to a lack of information of housing quality index at BAGA level, it was not possible to calculate the Diversity sub-score, consequently the maximum value for the composite scale (sum of all sub-scales except Diversity) was 60. Urbanicity scores were merged with the physical activity data by Basic Geographic Area (BAGA), so that for each participant an urbanicity score was given according to their household location.

### Statistical analysis

Moderate-to-vigorous physical activity (MVPA), sports activities, leisure time activities, PE class and active commuting to school were calculated as continuous variables by multiplying duration (minutes) per frequency (times per week) of the activities in the YPAQ. Due to violation of normality, these data were log-transformed. All urbanicity sub-scores were standardized in order to allow direct comparison between them and ensure their even contribution to the composite urbanicity scale.

To include information of all participants and increase statistical power, multiple imputation of missing data was implemented for 4,079 participants after confirming that the values were missing at random. Completeness of data was 82%. All physical activity measures (i.e., MVPA, sports activities, leisure time activities, PE class, active commuting to school), the six urbanicity sub-scores and participants’ characteristics that were potential predictors of missingness (i.e., sex, weight, height, parents’ education level, age, school, time they have been living in their latest address and state) were included in the multiple imputation model. Twenty imputed datasets were created using 20 cycles of regression switching and results were then averaged over these datasets using Rubin’s rules [31].

Descriptive statistics were calculated for all variables in the multiple imputed data set. A complete case analysis of the original dataset was also conducted to examine differences between estimates and is available in S2 Table. Body Mass Index (BMI) was computed by age and sex using weight and height of the participants and the Body Mass Index Cut-Offs for overweight and obese children (five to 19 years old) from the World Health Organization [32]. The associations between urbanicity and the five physical activity domains were examined using linear regression. The Wald test for an interaction between gender and the urbanicity-physical activity association revealed that including this variable improved (p<0.05) the fit of all the models. As such, linear regression models were performed for males and females separately to examine the association between urbanicity (exposure) and MVPA, sports activities, leisure activities, PE class, and active commuting (outcomes). The Wald test revealed there was evidence of an interaction by state (Mexico City vs. Oaxaca) and the association between overall urbanicity and sports activities. There was no evidence for further interactions by state. Twenty-four linear regression models were run, 20 regressions of physical activity variables (MVPA, sport activities, leisure activities, PE class, active commuting) and urbanicity (total and sub-scores) differentiating between sex, and four regressions of sport activities and urbanicity (total and sub-score) differentiating by state. All models were adjusted for parents’ education level, participants’ age and for school-level clustering using Robust Standard Errors. To facilitate comparison across variables, interpretation of results was based on the impact of a ½ standard deviation change in each variable. Analyses were performed using STATA, Version 13 (Statacorp, College Station, TX).

## Results

The final sample (N = 4079) from the imputed dataset consisted of 1,752 adolescents from Mexico City and 2,327 from Oaxaca. Forty-nine percent were males, mean age was 16.55±0.01 years and BMI was “normal” on average [32]. Descriptive statistics of participants’ parents’ education, BMI and physical activity by gender and state are presented in Tables 1, 2 and 3. Males reported higher MVPA (1,040.00 vs. 877.28 minutes per week), sports activity participation (731.07 vs. 573.15 minutes per week), time spent in PE class (15.83 vs 14.70 minutes per week) and active commuting (98.62 vs. 95.11 minutes per week) than females, while females reported more leisure physical activity (608.93 vs. 516.52 minutes per week). Participants living in Mexico City reported higher MVPA (1,025.47 vs. 910.56 minutes per week), leisure physical activity (625.80 vs. 514.83 minutes per week) and active commuting (129.40 vs. 72.11 minutes per week) than participants living in Oaxaca. Participants living in Oaxaca reported more time spent in PE class than those from Mexico City (20.16 vs. 8.83 minutes per week). Descriptive statistics for the unstandardized values of urbanicity sub-scores and scores by sex and state are reported in Table 4.

**Table 1:** Descriptive statistics of participants’ age and parents’ education level by gender and state.

	**Males (n = 2005)**		**Females (n = 2074)**	
**Age in years**	**n**	**%**	**n**	**%**
15	274	13.66	340	16.37
16	698	34.81	752	36.25
17	573	28.57	598	28.84
18	460	22.96	385	18.55
**Parents Education Level**	**n**	**%**	**n**	**%**
Primary School	155	7.73	257	12.37
Secondary School	559	27.88	573	27.62
College	725	36.18	740	35.69
Undergraduate Level	365	18.19	339	16.36
Postgraduate Degree	194	9.67	154	7.43
None	7	0.35	11	0.54
	**Mexico City (n = 1752)**		**Oaxaca (n = 2327)**	
**Age in years**	**n**	**%**	**n**	**%**
15	166	9.47	447	19.23
16	586	33.48	863	37.07
17	573	32.71	598	25.69
18	427	24.35	419	18.01
**Parents Education Level**	**n**	**%**	**n**	**%**
Primary School	101	5.76	310	13.32
Secondary School	477	27.23	652	28.00
College	723	41.29	744	31.95
Undergraduate Level	313	17.85	394	16.91
Postgraduate Degree	133	7.58	215	9.24
None	5	0.29	13	0.57

**Table 2:** Participants’ BMI per age by gender and state.

	**Males (n = 2005)**								**Females (n = 2074)**							
**Age**	**Un**		**No**		**Ov**		**Ob**		**Un**		**No**		**Ov**		**Ob**	
	**n**	**%**	**n**	**%**	**n**	**%**	**n**	**%**	**n**	**%**	**n**	**%**	**n**	**%**	**n**	**%**
15	5	1.8	157	57.2	87	31.8	25	9.1	7	1.9	246	72.4	69	20.3	18	5.4
16	17	2.4	437	62.6	201	28.8	43	6.1	17	2.1	553	73.6	159	21.2	23	3.1
17	10	1.8	395	68.9	130	22.6	38	6.7	14	2.3	449	75.1	121	20.2	14	2.4
18	10	2.3	315	68.4	108	23.4	27	5.9	4	1.2	298	77.3	65	16.9	18	4.5
	**Mexico City (n = 1752)**								**Oaxaca (n = 2327)**							
**Age**	**Un**		**No**		**Ov**		**Ob**		**Un**		**No**		**Ov**		**Ob**	
	**n**	**%**	**n**	**%**	**n**	**%**	**n**	**%**	**n**	**%**	**n**	**%**	**n**	**%**	**n**	**%**
15	2	1.5	99	59.7	48	28.9	16	9.8	9	2.1	306	68.3	109	24.4	23	5.2
16	13	2.3	410	69.9	142	24.3	21	3.5	20	2.3	586	67.9	220	25.6	37	4.3
17	9	1.6	420	73.4	118	20.6	25	4.4	15	2.5	428	71.5	134	22.4	21	3.6
18	10	2.2	254	59.6	138	32.2	25	5.9	5	1.3	263	62.7	117	27.9	34	8.1

Un: Underweight, No: Normal weight, Ov: Overweight, Ob: Obesity

**Table 3:** Participants’ physical activity by gender and state.

	**Males (n = 2005)**			**Females (n = 2074)**		
	**Mean**	**SD**	**Range**	**Mean**	**SD**	**Range**
Moderate to Vigorous Physical Activity (min/week) ^a^	1040.00	942.57	10.00–3360.00	877.28	894.69	10.00–3360.00
Sports Activities (min/week) ^a^	731.07	766.11	0.00–3360.00	573.15	708.92	0.00–3360.00
Leisure Activities (min/week) ^a^	516.52	564.90	0.00–3360.00	608.93	562.06	0.00–3360.00
PE Class (min/weekday) ^a^	15.83	59.48	0.00–900.00	14.70	49.07	0.00–700.00
Active Commuting (min/weekday) ^a^	98.62	175.81	0.00–1020.00	95.11	164.29	0.00–960.00
	**Mexico City (n = 1752)**			**Oaxaca (n = 2327)**		
	**Mean**	**SD**	**Range**	**Mean**	**SD**	**Range**
Moderate to Vigorous Physical Activity (min/week) ^b^	1025.47	986.11	10.00–3360.00	910.56	872.83	10.00–3360.00
Sports Activities (min/week) ^b^	656.15	788.47	0.00–3360.00	651.05	709.76	0.00–3360.00
Leisure Activities (min/week) ^b^	626.80	587.34	0.00–3360.00	514.83	543.60	0.00–3360.00
PE Class (min/weekday) ^b^	8.83	32.23	0.00–450.00	20.16	63.68	0.00–900.00
Active Commuting (min/weekday) ^b^	129.40	187.74	0.00–940.00	72.11	150.55	0.00–1020.00

^a^ T-test between males and females: p-value = 0.00

^b^ T-test between Mexico City and Oaxaca: p-value = 0.00

**Table 4:** Descriptive statistics of urbanicity variables by gender and state. Unstandardized values.

	Males (n = 2005)			Females (n = 2074)		
	Mean	SD	Range	Mean	SD	Range
Demographic*^a^	4.25	1.50	1.00–10.00	4.15	1.46	1.00–10.00
Economic Activity*^a^	4.32	0.47	1.87–5.99	4.35	0.47	1.87–7.09
Built Environment*^a^	8.74	0.86	5.5–10.00	8.75	0.88	1.00–10.00
Communication^a^	4.17	1.60	1.00–10.00	4.16	1.68	1.00–10.00
Education*^a^	7.03	0.72	3.80–9.44	7.06	0.76	3.93–9.75
Health*^a^	6.12	0.94	0.63–9.54	6.16	0.96	0.00–9.75
Overall^b^	34.65	2.04	25.29–45.49	34.66	2.05	23.29–44.42
	Mexico City (n = 1752)			Oaxaca (n = 2327)		
	Mean	SD	Range	Mean	SD	Range
Demographic*^a^	5.01	1.42	1.00–10.00	3.59	1.20	1.00–10.00
Economic Activity*^a^	4.55	0.40	2.76–7.09	4.17	0.45	1.87–5.62
Built Environment*^a^	8.96	0.56	7.00–10.00	8.59	1.02	1.00–10.00
Communication^a^	2.86	1.10	0.75–7.76	5.16	1.25	0.44–10.00
Education*^a^	7.35	0.74	4.19–9.75	6.82	0.66	3.80–9.43
Health*^a^	6.22	0.79	0.00–9.75	6.09	1.04	0.63–9.54
Overall^b^	34.96	1.85	23.29–44.36	34.43	2.11	25.29–45.49

^a^ Sub-score values range from 0 to 10.

^b^ Overall score value ranges from 0 to 60.

* T-test between males and females or Mexico City and Oaxaca: p-value<0.05

The multivariable regression models of overall urbanicity and urbanicity sub-scores by gender (Table 5) are presented by physical activity outcome. For MVPA, in the base model, every standard deviation increase in overall urbanicity was associated with a mean increase of 5% of females’ MVPA per week (95% CI = 0.00 to 0.11), there was no evidence of an association for males. In the multivariable linear regression model, for every standard deviation increase in the demographic sub-score there was a mean increase of 6% (95% CI = 0.01 to 0.10) MVPA per week for females. There was no evidence of an association between the other urbanicity sub-scores and MVPA.

**Table 5:** Linear regression models: Associations between urbanicity (z-scores), moderate to vigorous physical activity, sports activities, leisure activities, physical education class and active commuting. By gender.

Linear regression	Score/subscore	MVPA				Sports				Leisure Activities				PE Class				Active commuting			
		Min/wk^b^				Min/wk^b^				Min/wk^b^				Min/weekday^c^				Min/weekday^c^			
		Coef,	95% CI		P>|t|	Coef,	95% CI		P>|t|	Coef,	95% CI		P>|t|	Coef,	95% CI		P>|t|	Coef,	95% CI		P>|t|
**Males** **(n = 2005)** Base model^a^	Overall urbanicity	-0.01	-0.10	0.07	0.70	-0.04	-0.22	0.14	0.62	-0.08	-0.27	0.09	0.30	0.15	0.04	0.25	0.01	0.20	-0.00	0.42	0.05
Multivariable^a^	Demographic	-0.01	-0.09	0.07	0.79	-0.02	-0.15	0.09	0.61	-0.01	-0.21	0.17	0.85	0.01	-0.13	017	0.79	0.21	0.00	0.42	0.04
	Economic Activity	0.01	-0.06	0.08	0.75	-0.02	-0.23	0.18	0.78	0.00	-0.14	0.15	0.97	0.18	-0.10	0.47	0.17	-0.06	-0.23	0.09	0.37
	Built Environment	0.01	-0.05	0.08	0.64	0.02	-0.05	0.11	0.45	-0.02	-0.21	0.15	0.73	0.07	-0.00	0.16	0.07	0.08	-0.06	0.23	0.21
	Communication	-0.00	-0.12	0.11	0.93	0.23	0.00	0.46	0.04	-0.38	-0.71	-0.05	0.02	0.16	-0.29	0.61	0.43	-0.49	-0.91	-0.07	0.02
	Education	-0.05	-0.15	0.04	0.21	0.03	-0.18	0.25	0.70	-0.05	-0.39	0.28	0.71	-0.15	-0.45	0.13	0.24	-0.15	-0.49	0.18	0.30
	Health	-0.01	-0.08	0.04	0.57	-0.00	-0.16	0.16	0.97	-0.20	-0.37	-0.03	0.02	0.17	0.05	0.28	0.00	-0.01	-0.19	0.16	0.87
**Females (n = 2074)** Base model^a^	Overall urbanicity	0.05	0.00	0.11	0.04	-0.01	-0.14	0.12	0.85	0.02	-0.06	0.11	0.59	0.16	0.05	0.27	0.01	0.15	-0.04	0.35	0.11
Multivariable^a^	Demographic	0.06	0.01	0.10	0.01	-0.02	-0.12	0.08	0.62	0.06	0.00	0.12	0.04	0.01	-0.14	0.17	0.86	0.28	0.16	0.41	0.00
	Economic Activity	0.00	-0.05	0.06	0.75	0.04	-0.07	0.16	0.40	-0.11	-0.29	0.06	0.18	0.09	-0.03	0.21	0.14	-0.03	-0.20	0.13	0.63
	Built Environment	-0.04	-0.09	0.01	0.13	-0.07	-0.15	-0.00	0.04	-0.08	-0.21	0.04	0.18	0.07	-0.02	0.16	0.12	-0.08	-0.21	0.04	0.15
	Communication	-0.09	-0.24	0.06	0.19	0.08	-0.21	0.39	0.50	-0.36	-0.69	-0.03	0.03	0.08	-0.31	0.49	0.62	-0.40	-0.90	0.09	0.09
	Education	-0.02	-0.15	0.10	0.65	-0.01	-0.25	0.22	0.90	0.07	-0.20	0.35	0.54	-0.09	-0.36	0.17	0.44	-0.01	-0.36	0.33	0.92
	Health	0.04	-0.00	0.09	0.70	0.07	-0.06	0.22	0.23	-0.02	-0.13	0.09	0.62	0.22	0.06	0.38	0.01	-0.07	-0.20	0.05	0.24

^a^Adjusted by parents’ education level and participants’ age

^b^ Minutes per week (Monday to Sunday)

^c^ Minutes per weekday (Monday to Friday)

For sport participation, in the multivariable linear regression, for every standard deviation increase in the communication sub-score there was a mean increase of 23% (95% CI = 0.00 to 0.46) of time spent in sport activities in males. Amongst females, every standard deviation increase in the built environment sub-score was associated with 7% decrease (95% CI = -0.15 to -0.00) in sport participation.

For leisure physical activity, in the multivariable linear regression, amongst males there was a mean decrease of 38% (95% CI = -0.71 to -0.05) minutes per week for every standard deviation increase in the communication sub-score and mean decrease of 20% (95% CI = -0.37 to -0.03) for every standard deviation increase in the health sub-score. Amongst females, there was a mean decrease of 36% (95% CI = -0.69 to -0.03) minutes per week for every standard deviation increase in the communication sub-score and a mean increase of 6% (95% CI = 0.00 to 0.12) for every standard deviation increase in the demographic sub-score.

For time spent in PE class, in the linear regressions of the base models, there was a mean increase of 15% (95% CI = 0.04 to 0.25) in males and a 16% increase (95% CI = 0.05 to 0.27) in females for every standard deviation increase in overall urbanicity. In the multivariable linear regression, for every standard deviation increase in the health sub-score there was a mean increase of 17% (95% CI = 0.05 to 0.28) minutes per weekday in males and an increase of 22% (95% CI = 0.06 to 0.38) in females.

For active commuting, in the multivariable linear regression models, for every standard deviation increase in the demographic sub-score there was a mean increase of 21% (95% CI = 0.00 to 0.42) in males and an increase of 28% (95% CI = 0.16 to 0.41) in females, respectively. Additionally, for every standard deviation increase in the communication sub-score there was a mean decrease of 49% (95% CI = -0.91 to -0.07) minutes per weekday of active commuting in males.

Multivariable regression models of urbanicity and sport activities by state are presented in Table 6. Results are reported relative to the standard deviation units for the urbanicity values in Mexico City and Oaxaca.

**Table 6:** Linear regression models: Associations between urbanicity (z-scores) and sports activities. By state.

Linear regression	Score/subscore	Sports			
		Min/wk^b^			
		Coef,	95% CI		P>|t|
**Mexico City** **(n = 1752)** Base Model^a^	Overall urbanicity	0.16	0.02	0.31	0.03
Multivariable^a^	Demographic	0.07	0.01	0.14	0.02
	Economic Activity	-0.10	-0.32	0.11	0.26
	Built Environment	0.15	-0.09	0.40	0.16
	Communication	-0.05	-0.42	0.30	0.66
	Education	-0.17	-0.50	0.16	0.22
	Health	0.26	0.02	0.50	0.03
**Oaxaca (n = 2327)** Base Model^a^	Overall urbanicity	-0.09	-0.14	-0.03	0.01
Multivariable^a^	Demographic	-0.05	-0.11	0.00	0.05
	Economic Activity	0.07	-0.01	0.16	0.07
	Built Environment	-0.08	-0.12	-0.03	0.00
	Communication	-0.03	-0.40	0.33	0.77
	Education	-0.05	-0.35	0.23	0.55
	Health	-0.04	-0.15	0.05	0.26

^a^Adjusted by parents’ education level and participants’ age.

^b^ Minutes per week (Monday to Sunday)

In the base models, for every standard deviation increase in overall urbanicity there was a mean increase of 16% (95% CI = 0.02 to 0.31) in the time spent in sport activities of participants from in Mexico City and a mean decrease of 9% (95% CI = -0.14 to -0.03) in the time spent in sport activities of participants from Oaxaca. In the multivariable regression models, there was a mean increase of 7% (95% CI = 0.01 to 0.14) minutes per week with every standard deviation increase of the demographic sub-score and a mean increase of 26% (95% CI = 0.02 to 0.50) minutes per week per standard deviation increase in the health sub-score in Mexico. In Oaxaca, for every standard deviation increase in the built environment sub-score there was a mean decrease of 8% (95% CI = -0.12 to -0.03) time spent in sport activities.

## Discussion

This study investigated the association between five domains of physical activity and different indicators of urbanicity in adolescents from two contrasting states in Mexico; Mexico City which is 58.2 and Oaxaca which is comparatively 39.7. Household electronic media presence (assessed by the communication scale) was negatively associated with leisure time physical activity and active commuting in males. This finding is comparable to previous findings of a negative association between TV watching and time spent in leisure physical activity amongst Taiwanese adolescents [33]. There is no previous evidence that directly associates household electronic media presence and active commuting. However electronic media use is negatively associated with sleep quality [34–36], and adequate sleep duration is associated with greater active commuting to school [37].

Compared to adolescents from areas of low population density (demographic sub-score), adolescents who lived in areas of higher population density did more active commuting to school. Previous research has shown contrasting findings. Studies in Irish and German adolescents have found that living in more densely populated areas had greater odds of active commuting than those in less densely populated [38], and that living in rural areas was associated with lower levels of cycling compared to medium-sized towns [39]. In contrast, research in Mexico found that adolescents from urban areas were 32% less likely to engage in active commuting than their peers from rural areas [40]. The positive association between urbanicity defined by density and active commuting might be explained by the higher school concentration in highly populated areas, making it easier to walk or cycle to school. Also there might be a higher presence of walking trails or cycling routes that make it easier to commute [41]. On the other hand, the negative association previously found in Mexico [40] might be explained by the measurement of urbanicity an urban-rural dichotomy compared with our measure based on population density. An alternative explanation might be car ownership; with people dwelling in high density places more able to buy a car and primarily commute in that way [42]. More in-depth work is needed to further understand these associations.

Adolescents living in places with higher urbanicity and more health services spent more time in PE class. In line with our findings, children from urban areas in the United States reported a greater frequency of physical activity during PE class than children from rural areas [43]. We are not aware of evidence that supports the association between health services with time spent in PE class, and it might be the case that the health sub-score, which measures availability to health services that are more likely to be in more urbanised areas, is a proxy for broader urbanicity which may also reflect more well-developed PE provision in schools. Even though rural adolescents might be getting their physical activity from other sources, it is useful to identify PE as a potential setting in which to increase total physical activity in these areas of lower urbanicity.

Adolescents living in more urbanised areas of Mexico City spent more time in sport activities, compared to adolescents from more urbanised areas of Oaxaca. Adolescents in Mexico City living in a place with high urbanicity would spend 8% more time in sport activities than their peers living in a place with low urbanicity, while adolescents living in a place with high urbanicity in Oaxaca would spend 4.5% less time in sports than their peers living in a place with low urbanicity. Previous evidence coincides with the positive association found in Mexico City; for example, in Taiwan adolescents living in rural areas were less interested in recreational sports than adolescents living in urban areas [44]. Further, parents of children from urban schools were more likely to drive their children to sport facilities than parents from rural areas, and the weekly frequency of sports club attendance was higher amongst urban children [45]. This suggests that the direction of the association between overall urbanicity and participation in sport depends on the state. This could be explained by the density of sport facilities per square kilometre which is 0.01 in Oaxaca and 2.30 in, Mexico City [46]. In this instance, higher urbanicity may be supporting adolescent physical activity by providing access to places to do structured physical activity.

Among the strengths of the study is the use of a large data set which allowed various forms of physical activity to be examined. In addition, we sampled from a population that have not been fully studied in Mexico which was enhanced by use of a robust multiple imputation method. Further, we used a comprehensive measure of multidimensional urbanicity components that extend beyond the rural-urban dichotomy that has been used in other developing countries. Urbanicity data were derived using the most precise location level of data available (i.e., BAGA), which gives a better understanding of the environment in which each individual resides. Notwithstanding these strengths, the cross-sectional design prevents an understanding of the causal pathways at play in the urbanicity-physical activity association. Further, representativeness is limited to adolescents between 15 and 18 years old attending public schools as private schools were not sampled, also it is important to recognise the potential selection bias due to non-response to participate from 79 schools. Finally, schools from regions considered as unsafe in both states were excluded which may have implications for the variability of the urbanicity measure.

Understanding urbanicity and behaviours in the context in which they take place is key for the development of health programmes. The findings have implications at global and local level. Global physical activity guidelines should refer to the need to consider urbanicity in the physical activity plans of developing countries. Local health promotion teams should consider the nature and downstream effects of urbanicity such as increased communication infrastructure and access to media devices, in their local context.

## Conclusions

Urbanicity is related to young people’s physical activity in Mexico, however the associations are varied, with some components of urbanicity positively, and some negatively associated with different domains of physical activity. Our findings suggest that some urbanicity-physical activity associations may be different in places with different urbanicity. In Mexico City, areas with high urbanicity were associated with more time spent in PE class in males and females and more time spent in sport activities. In contrast, high urbanicity areas in Oaxaca were associated with less time spent in sport activities. The presence of electronic media at home (communication sub-score) was associated with less time spent in leisure physical activity in males and females and active commuting to school in males. High densely populated areas were associated with more active commuting.

## Supporting information

S1 Table YPAQ questionnaire with MET intensities and physical activity classification. (DOCX)

S2 Table Complete case analysis of the original dataset. (DOCX)
