# Supplementary material for: Association between urbanicity and physical activity in Mexican adolescents: The use of a composite urbanicity measure
Source: PLoS One. 2018 Sep 27;13(9):e0204739. doi: 10.1371/journal.pone.0204739 (PMC6160150; doi:10.1371/journal.pone.0204739)
Supplement: S1 Table — (DOCX) [file pone.0204739.s001.docx]

**YPAQ QUESTIONNAIRE WITH MET INTENSITIES AND PHYSICAL ACTIVITY CLASIFICATION**

| **LIST OF ACTIVITIES** | **MET VALUES CLASSIFICATION*** | | | **PHYSICAL ACTIVITY CLASSIFICATION** | | | | |
| --- | --- | --- | --- | --- | --- | --- | --- | --- |
|  | **CODE** | **METS** | **OBSERVATIONS** | **MVPA** | **SPORT**  **ACTIVITIES** | **LEISURE PA** | **PE CLASS** | **ACTITVE COMMUTING** |
| **SPORTS ACTIVITIES** |  |  |  |  |  |  |  |  |
| Aerobics | 03015 | 6.5 |  | X | X |  |  |  |
| Baseball/softball | 15620 | 5.0 |  | X | X |  |  |  |
| Basketball/volleyball | 15050 | 6.0 | Basket ball, non-game, general | X | X |  |  |  |
| Cricket | 15150 | 5.0 |  | X | X |  |  |  |
| Dancing | 03025 | 4.5 | General, Greek, Middle Eastern, hoola, flamenco, belly, swing | X | X |  |  |  |
| Football | 15610 | 7.0 | Casual, general soccer | X | X |  |  |  |
| Gymnastics | 15300 | 4.0 |  | X | X |  |  |  |
| Hockey (field or ice) | 15350 | 8.0 |  | X | X |  |  |  |
| Martial arts | 15430 | 10.0 |  | X | X |  |  |  |
| Netball | 15070 | 4.5 | Basketball, shooting baskets | X | X |  |  |  |
| Rugby | 15560 | 10.0 |  | X | X |  |  |  |
| American Futbol | 15560 | 10.0 | Same as rugby | X | X |  |  |  |
| Running or jogging | 12020 | 7.0 | Jogging, general | X | X |  |  |  |
| Swimming lessons | 18320 | 8.0 | Swimming sidestroke, general | X | X |  |  |  |
| Swimming for fun | 18310 | 6.0 | Swimming, leisurely, not lap swimming, general | X | X |  |  |  |
| Tennis/bádminton/squash/other racquet sport | 15675 | 7.0 | General | X | X |  |  |  |
| **LEISURE TIME ACTIVITIES** |  |  |  |  |  |  |  |  |
| Bike riding (not school travel) | 01015 | 8.0 | Bicycling, general | X |  | X |  |  |
| Trampolining | 15700 | 3.5 |  |  |  | X |  |  |
| Bowling | 15090 | 3.0 |  |  |  | X |  |  |
| Household chores | 05030 | 3.0 | Cleaning, house or cabin, general |  |  | X |  |  |
| Play on playground equipment | 15135 | 5.0 | Children’s games (dodege ball, hopscotch, 4-square, playground apparatus, t-ball, tetherball, marbles, acrace games) | X |  | X |  |  |
| Play with pets/horse riding | 05193  15370 | 4.0  4.0 | Walk/run, playing with animals, moderate, only active periods  Hoseback riding, general | X |  | X |  |  |
| Rollerblading/roller-skating | 15590 | 7.0 | Skating, roller | X |  | X |  |  |
| Scooter | 15580 | 5.0 | Same as skateboarding | X |  | X |  |  |
| Skateboarding | 15580 | 5.0 |  | X |  | X |  |  |
| Skiing, snowboarding, sledging | 19075 | 7.0 | Skiing, general, sledding | X |  | X |  |  |
| Skipping rope | 15552 | 8.0 | Rope jumping, slow | X |  | X |  |  |
| Walk the dog | 17165 | 3.0 |  |  |  | X |  |  |
| Walk for exercise/hiking | 17200  17080 | 3.8  6.0 | Walking 3.5 mphg, brisk, firm suface, walking for exercise.  Hiking, cross country |  |  | X |  |  |
| **ACTIVITIES AT SCHOOL** |  |  |  |  |  |  |  |  |
| Physical education class | 11876 | 6.5 | Teach physical education, exercise, sports clases(participate in the class) | X |  |  | X |  |
| Travel by walking to school (to and from school = 2 tiems) | 17270 | 4.0 | Walking, to work or class | X |  |  | X | X |
| Travel by cycling to school (to and from school = 2 times) | 01010 | 4.0 | Bicycling <10 mpg, leisure, to work or for pleasure. | X |  |  | X | X |
| **OTHER**  Please state:  ____________________ | MET values and physical activity classification was done according to each stated answer. | | | | | | | |
| MET: Metabolic Equivalent of Task  MVPA: Moderate-to-vigorous physical activity  PA: Physical Activity  PE: Physical Education  ** MET values from The Compendium of PA: and update of activity codes and MET intensities*  *http://www.juststand.org/portals/3/literature/compendium-of-physical-activities.pdf* | | | | | | | | |
