# Supplementary material for: Association between urbanicity and physical activity in Mexican adolescents: The use of a composite urbanicity measure
Source: PLoS One. 2018 Sep 27;13(9):e0204739. doi: 10.1371/journal.pone.0204739 (PMC6160150; doi:10.1371/journal.pone.0204739)
Supplement: S2 Table — (DOCX) [file pone.0204739.s002.docx]

Table 1. Descriptive Statistics of Participants’ Physical Activity by sex.

|  | Men | | | Women | | |
| --- | --- | --- | --- | --- | --- | --- |
|  | Mean | SD | Range | Mean | SD | Range |
| BMI(Kg/m^2^) by age: |  |  |  |  |  |  |
| 15 | 22.28 | 3.57 | 15.03-41.35 | 21.75 | 3.30 | 15.43-35.55 |
| 16 | 22.34 | 3.57 | 15.42-43.11 | 22.24 | 3.08 | 15.23-34.69 |
| 17 | 22.81 | 3.41 | 15.14-35.15 | 22.27 | 3.06 | 15.43-34.71 |
| 18 | 23.38 | 3.66 | 15.03-46.29 | 22.76 | 3.39 | 15.62-43.11 |
| Moderate to Vigorous Physical Activity (min/week)* | 917.26 | 795.78 | 10.00-3360.00 | 771.47 | 706.11 | 10.00-3360.00 |
| Sports Activities (min/week)* | 682.68 | 679.30 | 0.00-3360.00 | 422.24 | 457.40 | 0.00-3360.00 |
| Leisure Activities (min/week)* | 491.50 | 581.48 | 0.00-3360.00 | 593.07 | 586.42 | 0.00-3360.00 |
| PE Class (min/weekday)* | 13.34 | 59.72 | 0.00-900.00 | 12.25 | 48.64 | 0.00-700.00 |
| Active Commuting (min/weekday)* | 84.56 | 171.59 | 0.00-1020.00 | 81.60 | 157.97 | 0.00-960.00 |

* p-value=0.00

Table 2. Descriptive Statistics of Participants’ Physical Activity by state.

|  | Mexico City | | | Oaxaca | | |
| --- | --- | --- | --- | --- | --- | --- |
|  | Mean | SD | Range | Mean | SD | Range |
| BMI(Kg/m^2^) by age: |  |  |  |  |  |  |
| 15 | 22.59 | 3.78 | 15.03-41.35 | 21.74 | 3.26 | 15.23-34.02 |
| 16 | 22.16 | 3.21 | 15.23-34.23 | 22.40 | 3.41 | 15.42-43.11 |
| 17 | 22.52 | 3.17 | 15.14-33.20 | 22.58 | 3.31 | 15.43-35.15 |
| 18 | 22.99 | 3.30 | 15.62-36.22 | 23.24 | 3.80 | 15.03-46.29 |
| Moderate to Vigorous Physical Activity (min/week)* | 938.32 | 863.20 | 10.00-3360.00 | 847.14 | 751.400 | 10.00-3360.00 |
| Sports Activities (min/week)* | 555.54 | 607.32 | 0.00-3360.00 | 535.19 | 540.79 | 0.00-3360.00 |
| Leisure Activities (min/week)* | 608.34 | 625.89 | 0.00-3360.00 | 495.22 | 552.63 | 0.00-3360.00 |
| PE Class (min/weekday)* | 5.19 | 29.09 | 0.00-450.00 | 18.30 | 64.25 | 0.00-900.00 |
| Active Commuting (min/weekday)* | 112.23 | 183.96 | 0.00-940.00 | 61.73 | 145.10 | 0.00-1020.00 |

* p-value=0.00

Table 3. Descriptive Statistics of Urbanicity Variables in men and women. Unstandardized values.

|  | Men | | | Women | | |
| --- | --- | --- | --- | --- | --- | --- |
|  | Mean | SD | Range | Mean | SD | Range |
| Demographic* | 4.32 | 1.62 | 1.00-10.00 | 4.20 | 1.55 | 1.00-10.00 |
| Economic Activity* | 4.32 | 0.47 | 1.87-5.99 | 4.35 | 0.47 | 1.87-7.09 |
| Built Environment* | 8.7 | 0.86 | 5.5-10.00 | 8.76 | 0.88 | 1.00-10.00 |
| Communication* | 4.10 | 1.57 | 0.33-7.80 | 4.09 | 1.67 | 0.30-8.64 |
| Education* | 6.9 | 0.66 | 4.68-9.14 | 7.00 | 0.70 | 5.25-9.22 |
| Health* | 6.12 | 0.94 | 0.63-9.54 | 6.16 | 0.96 | 0.00-9.75 |
| Overall* | 38.66 | 3.37 | 26.73-47.94 | 38.71 | 3.42 | 26.73-47.94 |

^a^ Sub-score values range from 0 to 10 and overall from 0 to 60.

*p-value<0.05

Table 4. Descriptive Statistics of Urbanicity Variables in Mexico City and Oaxaca. Unstandardized values.

|  | Mexico City | | | Oaxaca | | |
| --- | --- | --- | --- | --- | --- | --- |
|  | Mean | SD | Range | Mean | SD | Range |
| Demographic* | 5.01 | 1.42 | 1.00-10.00 | 3.59 | 1.20 | 1.00-10.00 |
| Economic Activity* | 4.55 | 0.40 | 2.76-7.09 | 4.17 | 0.45 | 1.87-5.62 |
| Built Environment* | 8.96 | 0.56 | 7.00-10.00 | 8.59 | 1.02 | 1.00-10.00 |
| Communication | 2.86 | 1.10 | 0.30-6.65 | 5.14 | 1.20 | 0.44-8.64 |
| Education* | 7.26 | 0.71 | 5.75-9.22 | 6.75 | 0.56 | 4.68-9.14 |
| Health* | 6.22 | 0.79 | 0.00-9.75 | 6.09 | 1.05 | 0.63-9.54 |
| Overall | 41.10 | 2.35 | 30.66-47.94 | 36.60 | 2.71 | 26.73-42.33 |

^a^ Sub-score values range from 0 to 10 and overall from 0 to 60.

*p-value<0.05

Table 5. Linear Regression Models: Associations Between Urbanicity (Z-scores), Moderato to Vigorous Physical Activity, Sports Activities, Leisure Activities, Physical Education Class and Active Commuting. By sex.

|  |  | **MVPA** | | | | **Sports** | | | | **Leisure Activities** | | | | **PE Class** | | | | **Active commuting** | | | |
| --- | --- | --- | --- | --- | --- | --- | --- | --- | --- | --- | --- | --- | --- | --- | --- | --- | --- | --- | --- | --- | --- |
|  |  | **Min/wk** | | | | **Min/wk** | | | | **Min/wk** | | | |  |  |  |  |  |  |  |  |
| **Linear regression** | **Score/subscore** | **Coef,** | **95% CI** | | **P>\|t\|** | **Coef,** | **95% CI** | | **P>\|t\|** | **Coef,** | **95% CI** | | **P>\|t\|** | **Coef,** | **95% CI** | | **P>\|t\|** | **Coef,** | **95% CI** | | **P>\|t\|** |
| **Men**  Simple | Overall urbanicity | -0.12 | -0.19 | -0.05 | 0.00 | -0.26 | -0.40 | -0.12 | 0.00 | 0.12 | -0.12 | 0.38 | 0.27 | 0.10 | 0.04 | 0.21 | 0.02 | 0.41 | 0.28 | 0.55 | 0.00 |
| Multivariable | Demographic | -0.03 | -0.12 | 0.05 | 0.36 | -0.04 | -0.26 | 0.17 | 0.63 | 0.00 | -0.26 | 0.27 | 0.97 | 0.00 | -0.10 | 0.11 | 0.90 | 0.26 | 0.05 | 0.48 | 0.01 |
|  | Economic Activity | 0.03 | -0.03 | 0.10 | 0.33 | -0.03 | -0.23 | 0.16 | 0.68 | 0.05 | -0.21 | 0.32 | 0.66 | 0.12 | -0.02 | 0.28 | 0.09 | -0.16 | -0.36 | 0.04 | 0.11 |
|  | Built Environment | -0.08 | -0.16 | -0.00 | 0.03 | 0.01 | -0.11 | 0.15 | 0.79 | -0.08 | -0.25 | 0.07 | 0.25 | 0.04 | -0.00 | 0.10 | 0.08 | 0.12 | -0.03 | 0.28 | 0.11 |
|  | Communication | 0.07 | -0.06 | 0.21 | 0.23 | 0.34 | 0.09 | 0.58 | 0.01 | -0.33 | -0.73 | 0.06 | 0.08 | 0.12 | -0.24 | 0.48 | 0.47 | -0.46 | -0.94 | -0.06 | 0.05 |
|  | Education | 0.00 | -0.05 | 0.06 | 0.91 | 0.08 | -0.05 | 0.21 | 0.21 | 0.03 | -0.30 | 0.37 | 0.81 | -0.10 | -0.31 | 0.11 | 0.30 | 0.00 | -0.27 | 0.28 | 0.95 |
|  | Health | -0.04 | -0.13 | 0.04 | 0.25 | 0.00 | -0.22 | 0.23 | 0.97 | -0.32 | -0.53 | -0.11 | 0.00 | 0.16 | 0.01 | 0.31 | 0.03 | -0.11 | -0.40 | 0.17 | 0.39 |
| **Women**  Simple | Overall urbanicity | 0.03 | 0.02 | 0.09 | 0.02 | -0.10 | -0.20 | -0.00 | 0.04 | 0.18 | -0.06 | 0.44 | 0.12 | 0.06 | -0.14 | 0.26 | 0.50 | 0.33 | 0.10 | 0.56 | 0.01 |
| Multivariable | Demographic | 0.02 | 0.01 | 0.05 | 0.00 | -0.13 | -0.25 | -0.00 | 0.04 | 0.02 | 0.00 | 0.20 | 0.05 | 0.01 | -0.06 | 0.09 | 0.75 | 0.21 | 0.12 | 0.45 | 0.04 |
|  | Economic Activity | 0.00 | -0.07 | 0.08 | 0.98 | 0.05 | -0.08 | 0.19 | 0.36 | -0.22 | -0.39 | -0.05 | 0.01 | 0.03 | -0.17 | 0.24 | 0.73 | -0.03 | -0.25 | 0.18 | 0.74 |
|  | Built Environment | -0.09 | -0.14 | -0.04 | 0.00 | -0.13 | -0.23 | -0.02 | 0.01 | -0.08 | -0.18 | 0.02 | 0.11 | 0.09 | 0.01 | 0.17 | 0.03 | -0.03 | -0.16 | 0.10 | 0.613 |
|  | Communication | -0.07 | -0.23 | 0.08 | 0.30 | -0.01 | -0.25 | 0.21 | 0.86 | -0.39 | -0.66 | -0.13 | 0.00 | 0.13 | -0.21 | 0.49 | 0.40 | -0.29 | -0.86 | 0.27 | 0.27 |
|  | Education | -0.00 | -0.10 | 0.10 | 0.99 | -0.06 | -0.22 | 0.10 | 0.41 | 0.14 | -0.16 | 0.45 | 0.32 | -0.04 | -0.21 | 0.11 | 0.54 | 0.08 | -0.17 | 0.35 | 0.46 |
|  | Health | 0.00 | -0.07 | 0.09 | 0.81 | 0.07 | -0.07 | 0.21 | 0.30 | -0.16 | -0.36 | 0.03 | 0.09 | 0.31 | 0.01 | 0.61 | 0.03 | -0.15 | -0.46 | 0.14 | 0.26 |

^a^Adjusted by parents’ education level and participants’ age

Table 6. Linear Regression Models: Associations Between Urbanicity (Z-scores) and Sports Activities. By state.

|  |  | **Sports** | | | |
| --- | --- | --- | --- | --- | --- |
|  |  | **Min/wk** | | | |
| **Linear regression** | **Score/subscore** | **Coef,** | **95% CI** | | **P>\|t\|** |
| **Mexico City**  Simple | Overall urbanicity | 0.10 | 0.02 | 0.42 | 0.04 |
| Multivariable | Demographic | 0.02 | -0.01 | 0.06 | 0.06 |
|  | Economic Activity | -0.11 | -0.24 | 0.02 | 0.08 |
|  | Built Environment | 0.10 | -0.23 | 0.44 | 0.48 |
|  | Communication | -0.14 | -0.46 | 0.17 | 0.30 |
|  | Education | -0.14 | -0.27 | -0.01 | 0.03 |
|  | Health | 0.25 | 0.03 | 0.43 | 0.02 |
| **Oaxaca**  Simple | Overall urbanicity | -0.12 | -0.24 | -0.00 | 0.04 |
| Multivariable | Demographic | -0.11 | -0.21 | -0.01 | 0.03 |
|  | Economic Activity | 0.12 | 0.10 | 0.14 | 0.00 |
|  | Built Environment | -0.11 | -0.20 | -0.03 | 0.02 |
|  | Communication | -0.06 | -0.39 | 0.26 | 0.61 |
|  | Education | -0.08 | -0.27 | 0.09 | 0.25 |
|  | Health | -0.06 | -0.10 | -0.02 | 0.00 |

*Adjusted by parents’ education level and participants’ age.
